# Ethanol Sclerotherapy in the Management of Ovarian Endometrioma: Technical Considerations for Catheter- and Needle-Directed Sclerotherapy

**DOI:** 10.1007/s00270-024-03694-0

**Published:** 2024-03-29

**Authors:** Aynur Azizova, Turkmen Turan Ciftci, Murat Gultekin, Emre Unal, Okan Akhan, Gurkan Bozdag, Devrim Akinci

**Affiliations:** 1 Department of Radiology, Hacettepe University School of Medicine, 06100 Sihhiye, Ankara Turkey; 2 Department of Obstetrics and Gynecology, Hacettepe University School of Medicine, 06100 Sihhiye, Ankara Turkey

**Keywords:** Endometriosis, Infertility, Dysmenorrhea, Ethanol sclerotherapy, Ovarian reserve, AMH

## Abstract

**Purpose:**

To provide technical guidance on applying catheter-directed and needle-directed ethanol sclerotherapy for endometriomas and present the results of these sclerotherapy methods.

**Materials and Methods:**

From January 2015 to March 2021, the results of the patients with symptomatic ovarian endometriomas who underwent needle-directed or catheter-directed sclerotherapy were evaluated, retrospectively. The decision to apply which sclerotherapy technique was made during the procedure for each patient considering the following factors: cyst size, cyst location, cyst viscosity, and tissue rigidity.

**Results:**

Both needle-directed (*n* = 34 cysts) and catheter-directed (*n* = 34 cysts) sclerotherapy techniques were effective, with a 100% technical success rate and a 97% clinical success rate. In two of 34 cysts (6%) treated with needle-directed sclerotherapy, recurrence was detected and successfully retreated with catheter-directed sclerotherapy. Significant reductions in cyst size, pain, and serum cancer antigen 125 levels (*p* < 0.05) were noted. Serum anti-Müllerian hormone levels remained unaffected, indicating preserved ovarian reserve (*p* > 0.05). Among those treated for infertility, the pregnancy rate was 54% (*n* = 6/11). The mean ± SD cyst size decline was greater in catheter-directed sclerotherapy than needle-directed sclerotherapy (5.5 ± 3.1 cm vs. 4.0 ± 2.1 cm, *p* < 0.05). However, the pretreatment cyst volumes were considerably higher in catheter-directed sclerotherapy group (202.0 ± 233.5 mL vs. 78.8 ± 59.7 mL, *p* < 0.05) and were associated with significant post-treatment volume decrease (*p* < 0.05).

**Conclusion:**

The choice between catheter-directed and needle-directed ethanol sclerotherapy should be determined during the procedure, with a preference for catheter-directed sclerotherapy when feasible. Crucial factors in making this decision include cyst size, cyst location, cyst viscosity, and tissue rigidity.

Level of evidence Level 3, non-controlled retrospective cohort study.

**Graphical Abstract:**

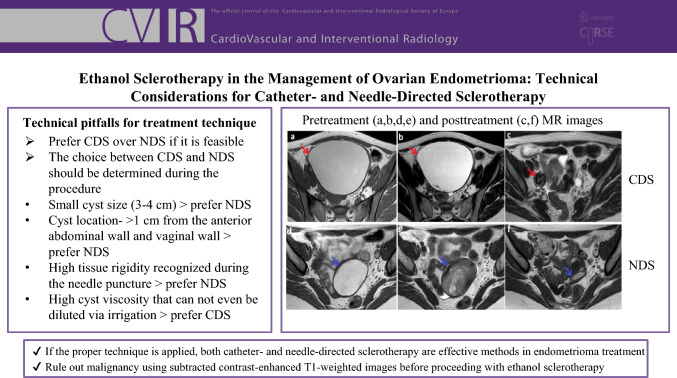

**Supplementary Information:**

The online version contains supplementary material available at 10.1007/s00270-024-03694-0.

## Introduction

Endometriosis is an estrogen-dependent chronic benign disease occurring due to the endometrial tissue existence in the extrauterine environment and affecting approximately 10% of women of reproductive age [1]. Endometrioma is the most common form of pelvic endometriosis, characterized by a cystic lesion with the wall consisting of endometrial mucosa occurring following recurrent hemorrhages. Patients with endometriosis frequently complain of dysmenorrhea, chronic pelvic pain, or dyspareunia. Additionally, endometriosis is associated with diminished ovarian reserve and infertility. The reported endometriosis frequency in infertile women is approximately 25–50% [1–5].

Ovarian endometrioma treatment aims to treat life quality reducing symptoms, such as dysmenorrhea, and preserve ovarian reserve by minimizing ovarian injury. Although surgical excision is the standard treatment method, the depreciation in ovarian reserve owing to removing healthy ovarian tissue adjacent to endometrioma or electrocoagulation is inevitable. Although oral contraceptives are used for the treatment, their utilization is limited due to high recurrence rates and side effects such as thromboembolism [4–7].

Ethanol sclerotherapy applied to treat benign cystic lesions of solid organs has also been used to treat endometrioma, primarily due to its minimally invasive feature. It has been shown that ethanol sclerotherapy preserves ovarian reserve due to the precise targeting endometrioma without causing normal ovarian tissue damage. Moreover, a marked decrease in the cyst size dissolves the mass effect on the ovary and is associated with better ovarian reserve [4, 5]. A recent meta-analysis assessing the effectiveness of ultrasound-guided sclerotherapy for endometrioma concluded that it is a safe and efficient method for managing recurrence, infertility, and pain [8].

Ethanol sclerotherapy can be performed via a needle [9] or a catheter [4]. Some drawbacks of needle-directed sclerotherapy (NDS) compared to catheter-directed sclerotherapy (CDS) include difficulties in effectively evacuating viscous endometrioma content with a 16–18-gauge needle, potential needle dislodgement, leading to leakage of cyst contents and peritoneal adhesions, and decreased treatment efficacy. NDS for multiloculated lesions is also technically challenging, resulting in inadequate cyst content evacuation and reduced treatment efficacy. Moreover, recurrence rates after NDS range from 0 to 62% in the literature. [4]. However, the technical considerations for patient selection in choosing between CDS or NDS remain unclear.

This study aimed to provide interventional radiologists with technical guidance on applying ethanol sclerotherapy for endometriomas by elucidating when to perform the procedure through a catheter or a needle based on our single-center experience and presenting the results of these sclerotherapy methods.

## Materials and Methods

This retrospective observational descriptive study was approved by the institutional review board and designed following the STrengthening and Reporting of OBservational studies in Epidemiology (STROBE) guidelines. Picture archiving and communication systems with electronic medical records were searched to collect patients' data from January 2015 to March 2021. All patients with ovarian endometrioma treated with ethanol sclerotherapy due to pain or infertility complaints were included consecutively. Patients lost to follow-up were excluded.

All patients were evaluated with ultrasound and contrast-enhanced magnetic resonance imaging (CE-MRI) to confirm endometrioma diagnosis and exclude malignancy. Obtaining subtraction images of pre-contrast T1-weighted images from post-contrast T1-weighted images was mandatory to rule out malignancy. Complaints such as pain and infertility were questioned, and Visual Analogue Scale (VAS) scores were recorded. Serum cancer antigen 125 (CA-125) and serum anti-Müllerian hormone (AMH) levels were measured.

The treatment decision was taken after evaluation of the patient in the multidisciplinary team forum involving at least one interventional radiologist and a gynecologist. The preferred primary treatment method for ovarian endometrioma was ethanol sclerotherapy, aiming to preserve ovarian reserve. Inclusion criteria for sclerotherapy were (i) cysts concordant with endometrioma, (ii) cysts without the sign of malignancy such as a solid enhancing component according to CE-MRI, (iii) maximum cyst diameter larger than 3 cm, (iv) symptomatic cysts associated with pain or infertility, (v) the presence of the access to the cyst via the transabdominal or transvaginal route. Exclusion criteria for sclerotherapy were (i) cysts with the sign of malignancy and (ii) the absence of the transabdominal access and inability to use the transvaginal route due to virginity. Surgery was considered only for these excluded cases.

All patients were treated as inpatients after obtaining informed consent. Coagulation parameters (platelet count > 50,000/μL and international normalized ratio < 1.2) were determined. All procedures were performed by one of three interventional radiologists (E.U., T.T.C., D.A.) who had at least five years of experience. Procedures were performed in an interventional radiology unit equipped with fluoroscopy and ultrasound in the supine or lithotomy position under sterile conditions. Intravenous sedation was administered by the anesthesiologist using midazolam (0.05–0.1 mg/kg), fentanyl (0.5–1 µg/kg), and propofol (0.5–1 mg/kg).

### Treatment Techniques: Selection Criteria

The choice between CDS or NDS technique was determined during the procedure for each patient, taking into account the following factors collectively: (i) cyst size—small cysts with maximum diameter between 3 and 4 cm were treated with NDS as catheter placement in small cysts increase the risk of rupture; CDS was preferred for the cysts > 4 cm, (ii) cyst location—cysts located more than 1 cm away from the anterior abdominal or vaginal wall, with intraabdominal tissues like bowel loops or paraovarian vascular structures in between, were treated with NDS; otherwise, CDS was the preferred approach, (iii) cyst viscosity—if the viscosity of endometrioma content was high that cannot even be diluted via irrigation, CDS was preferred over NDS, (iv) tissue rigidity—if the rigidity of tissues, especially vaginal wall, recognized during the needle puncture was high, the technique of choice was NDS as catheter placement could increase the rupture risk. Treatments were performed using transabdominal or transvaginal access. Cysts with the possibility of direct access from the anterior abdominal wall were treated via transabdominal route, and otherwise, transvaginal access was preferred. All multiloculated cysts were treated with CDS. Patients with multiple cysts were treated in the same session.

### Needle-Directed Sclerotherapy Technique

**Fig. 1:**
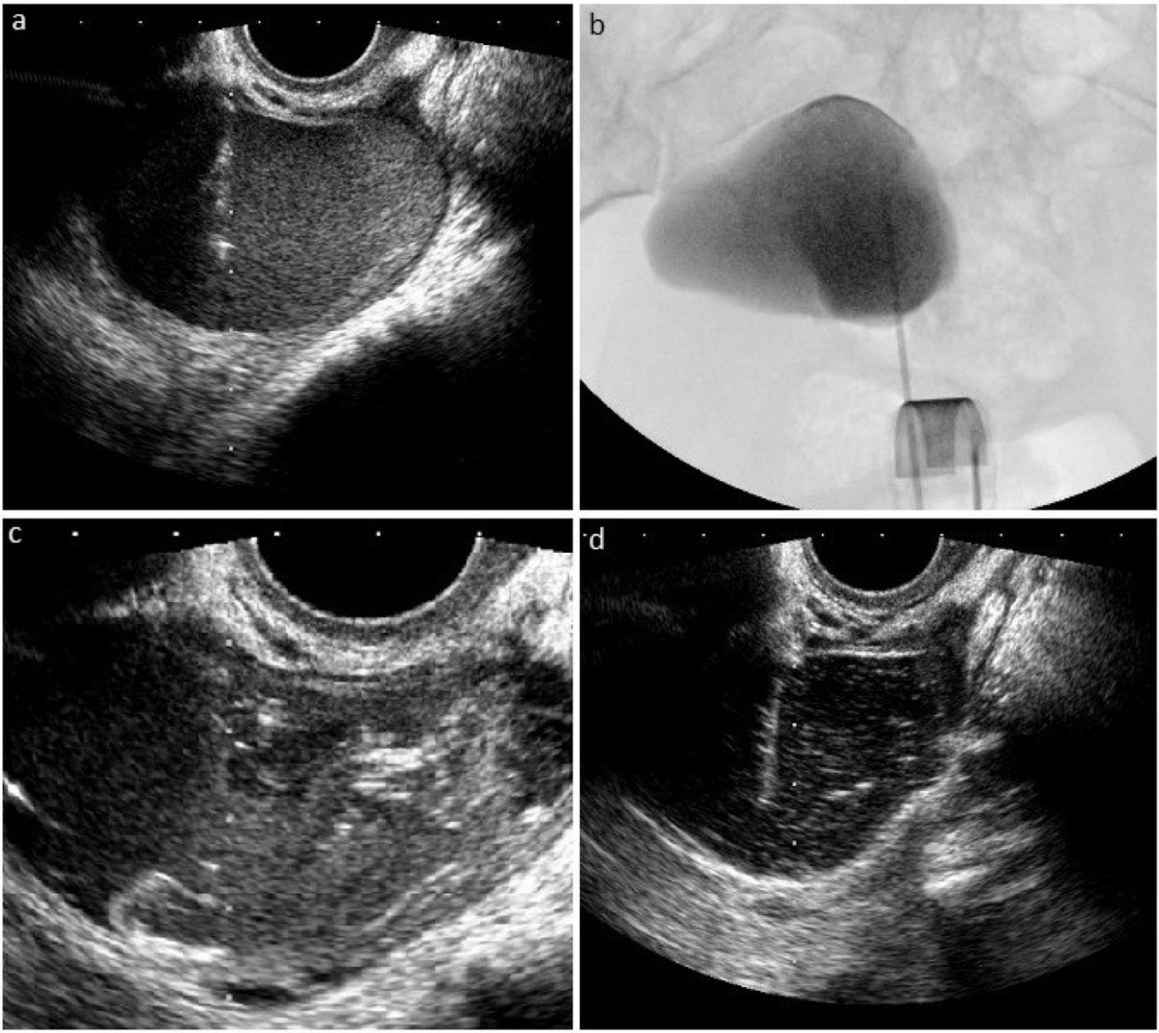
NDS technique. **a** Under transvaginal ultrasound guidance, right ovarian endometrioma was punctured using an 18-gauge Chiba needle. **b** After aspiration of roughly 20% of the estimated cyst volume, the contrast agent less than the aspirated content was injected under fluoroscopic guidance to verify the absence of leakage. **c** Next, the viscosity of the cyst content was reduced by irrigation with sterile saline injections and aspirations. **d** After the cyst content turned serous, sclerotherapy was applied with sterile 96% ethanol for 15 min. The procedure was terminated after the reaspiration of the ethanol. NDS = needle-directed sclerotherapy

The cyst was punctured using an 18-gauge Chiba needle under sonographic guidance (Fig. 1a). Approximately 20% of the estimated cyst volume was aspirated; subsequently, the contrast agent (Ultravist 300/100 mg/mL; Bayer, Leverkusen, Germany), less than the aspirated content, was injected under fluoroscopic guidance to confirm the leakage absence (Fig. 1b). After that, irrigation with 3–5 mL sterile saline injections and aspirations was performed to reduce the viscosity of the hemorrhagic cyst content (Fig. 1c). When the cyst content became completely serous, the remaining content was aspirated almost entirely by keeping the tip of the needle within the cavity. Eventually, sclerotherapy was performed with sterile 96% ethanol (50% of the estimated volume, not to exceed 100 mL) for 15 min (Fig. 1d). Finally, the procedure was terminated after the reaspiration of the ethanol.

### Catheter-Directed Sclerotherapy Technique

**Fig. 2:**
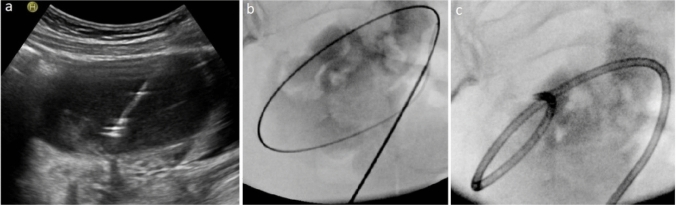
CDS technique. **a** Under transabdominal ultrasound guidance, the left ovarian endometrioma was punctured with an 18-gauge needle. **b** Then, a 0.035-inch Amplatz guidewire was advanced into the cyst under ultrasound and fluoroscopy guidance through the needle, and **c** an 8-F drainage catheter was placed. After the evacuation of the cyst content and obtaining a cystogram to verify the absence of leakage, sclerotherapy was applied with sterile 96% ethanol for 15 min. Finally, the procedure was terminated after the reaspiration of the ethanol. CDS = catheter-directed sclerotherapy

After puncturing the cyst with an 18-gauge needle (Fig. 2a), a 0.035-inch Amplatz guidewire (Boston Scientific, USA) was advanced into the cyst under ultrasound and fluoroscopy guidance through the needle (Fig. 2b). In the presence of the multilocular cyst, the internal septa were mechanically fragmented with a 0.035-inch guidewire and dilator manipulation. Next, an 8-F drainage catheter (Skater, Argon Medical Devices, USA) was placed (Fig. 2c). After the cyst content evacuation and obtaining a cystogram to confirm the leakage absence, sclerotherapy was performed with sterile 96% ethanol for 15 min. The catheter was withdrawn after the evacuation of the entire ethanol content, and the procedure was terminated (Supplementary Video 1).

### Post-Procedural Care

The aspirated cyst content was sent for cytological examination. All patients were monitored in the recovery area for 1 h and transferred to inpatient hospitalization. They were discharged after 4–6 h of follow-up or the following day if they had normal vital signs. Complications related to the procedure were recorded. Patients were followed up with ultrasound and/or MRI 3 and 6 months after the procedure and annually, thereafter. In addition, VAS scores, serum CA-125, and AMH levels were evaluated during each follow-up.

### Definitions and Statistical Analysis

Technical success was defined as accomplishing all procedure steps without any intraprocedural complication. Clinical success was defined as the reduction or disappearance of cysts, decline (VAS score to 1–3 range), or disappearance (VAS = 0) of pain in follow-up. Clinical failure was defined as an increase or no decrease in cyst size and complaints. An increase in cyst size was considered as recurrence. The cyst volumes were calculated using the ellipsoid formula (largest three axes × 0.523) on ultrasound and MRI. The serum CA-125 and serum AMH levels before the treatment and at the last follow-up were evaluated. Complications were defined using the Cardiovascular and Interventional Radiological Society of Europe (CIRSE) classification for complications [10]. The degree of pain before and after the treatment was determined from 0 to 10 points using VAS [11, 12].

All statistical analyses were conducted using SPSS 11.5 (IBM) software. Quantitative variables were described using mean ± standard deviation or median (minimum–maximum), while qualitative variables were described using the number of patients/cysts (percentage). Student's t-test or Mann–Whitney U test was used to compare quantitative variables between two categories of qualitative variables, depending on normal distribution assumptions. For qualitative variables with more than two categories, one-way ANOVA or Kruskal–Wallis H test was applied based on normal distribution assumptions. Chi-square and Fisher-exact tests examined the relationship between two qualitative variables. Differences between two quantitative dependent variables were assessed using the paired t-test or Wilcoxon sign test, depending on normal distribution assumptions. A *p*-value of < 0.05 was considered statistically significant.

## Results

Fifty-one consecutive patients (main complaint: pain *n* = 40, infertility *n* = 11) with 68 cysts treated with ethanol sclerotherapy were included in this study. Four patients with four cysts were excluded due to loss to follow-up. Table 1 summarizes the baseline characteristics of participants.

**Table 1:** Baseline characteristics of patients and cysts

Characteristics	Values
*Age (year)*	
Mean + SD	30.0 ± 5.8
Median (Min–Max.)	30.0 (15.0–40.0)
*Cysts per patient*	
Mean + SD	1.3 ± 0.6
Median (Min–Max.)	1.0 (1.0–4.0)
*Location of cysts, n(%)*	
Unilateral	39.0 (76.0)
Bilateral	12.0 (24.0)
*Morphology of cysts, n(%)*	
Unilocular	61.0 (90.0)
Multilocular	7.0 (10.0)
*Main symptom, n(%)*	
Pain	40.0 (78.0)
Infertility	11.0 (22.0)
*Oral contraceptive use before treatment, n(%)*	
Yes	3.0 (6.0)
No	48.0 (94.0)
*History of surgery for endometrioma before treatment, n(%)*	
Yes	1.0 (2.0)
No	50.0 (98.0)
*Treatment technique, n(%)*	
CDS	34.0 (50.0)
NDS	34.0 (50.0)
*Treatment route, n(%)*	
Transabdominal	39.0 (57.0)
Transvaginal	29.0 (43.0)
*Treatment-related complication, n(%)*	
Yes	1.0 (2.0)
No	50.0 (98.0)
*Hospitalization days*	
Mean + SD	0.7 ± 0.4
Median (Min.–Max.)	1.0 (0.0–1.0)
*Follow-up periods (months)*	
Mean + SD	14.5 ± 11.0
Median (Min.-Max.)	14.0 (1.0–55.0)
*Pain relief in patients who treated for pain, n(%)*	
Yes	40.0 (100.0)
No	0.0 (0.0)
*Post-treatment pregnancy who treated for infertility, n(%)*	
Yes	6.0 (54.0)
No	5.0 (46.0)

*SD* Standard deviation, *Min* Minimum, *Max* Maximum, *CDS* Catheter-directed sclerotherapy, *NDS* Needle-directed sclerotherapy

The technical success rate was 100%. CDS and NDS were used to treat 34 (50%) and 34 (50%) cysts, respectively. All aspirates were confirmed to contain hemosiderin-laden macrophages compatible with endometrioma and were negative for malignancy. The mean ± SD length of hospital stay was 0.7 ± 0.4 days. The mean ± SD follow-up was 14.5 ± 11.0 months (range: 1.0–55.0 months). The only complication (grade 3 [10]) was cavity infection observed in one patient (2%) treated with NDS via vaginal approach. Fifteen days after the procedure, the patient was admitted to the emergency department with a high fever, raising suspicion of cavity infection. A sample was taken from the treated residual cyst cavity via vaginal needle aspiration, showing a negative culture for infection. Considering no other cause for the fever, it was attributed to a procedure-related complication and treated with intravenous antibiotics, leading to resolution and subsequent discharge of the patient.

The clinical success rate was 97%. The recurrence rate was 3%. One patient with two cysts, initially treated with NDS for infertility, necessitated retreatment due to recurrence, with cyst volumes increasing by 70% nine months post-procedure. Following the second session with CDS, there was a remarkable 98% reduction in cyst volumes nine months after the subsequent procedure.

**Fig. 3:**
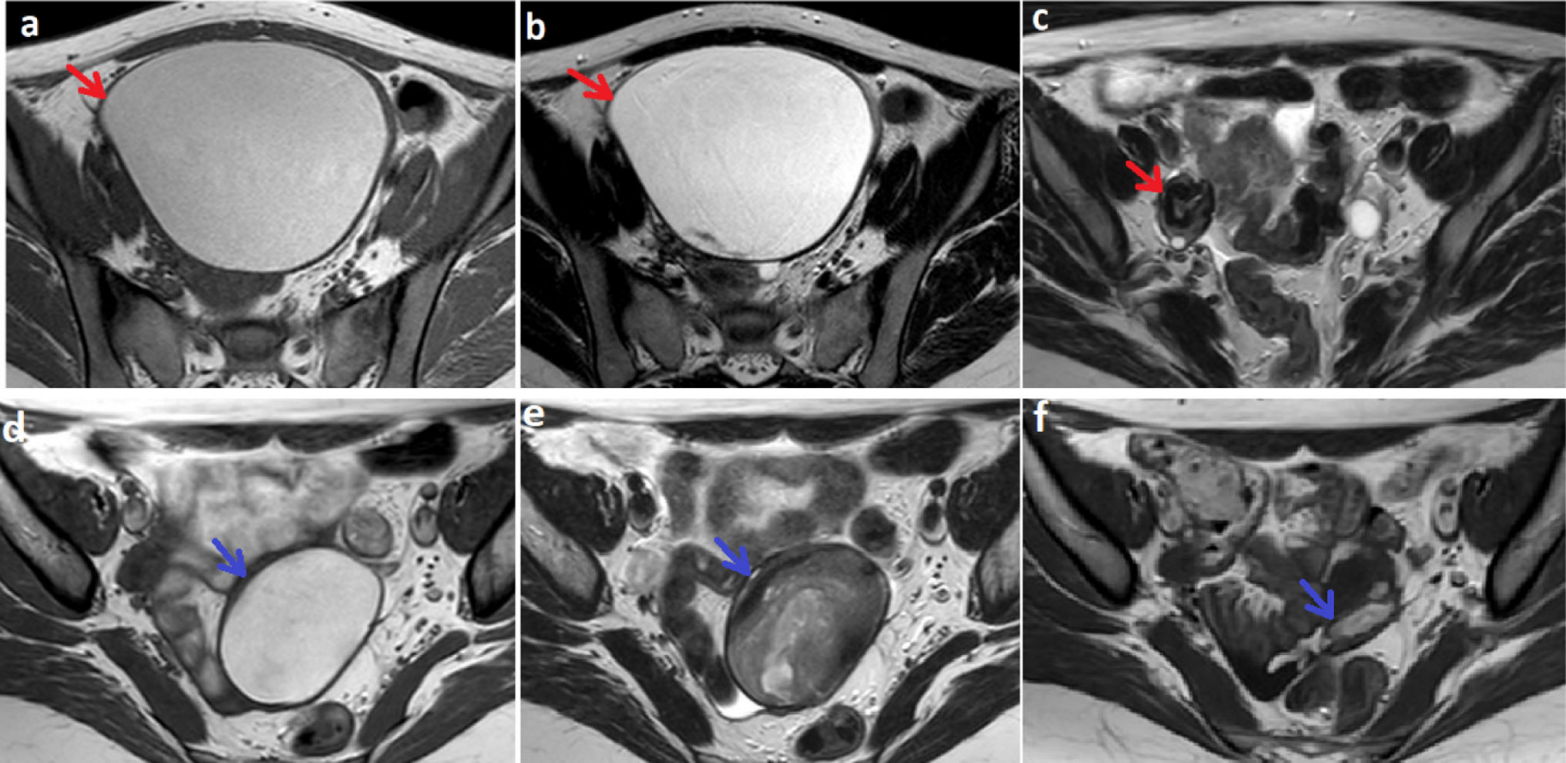
The pretreatment and posttreatment images of two different patients treated with ethanol sclerotherapy. **a**, **b**, **c** A 27-year-old patient with right ovarian endometrioma complaining of pain was treated with CDS. In the 54-months follow-up, the huge cyst (transverse T1W(**a**)/T2W(**b**) images, red arrows) disappeared (c- transverse T2W image, red arrow). **d**, **e**, **f** A 37-year-old patient with left ovarian endometrioma was treated for infertility with NDS. In the 16-months follow-up, the cyst (transverse T1W(**d**)/T2W(**e**) images, blue arrows) regressed almost completely (transverse T2W(**f**) image, blue arrow)

**Fig. 4:**
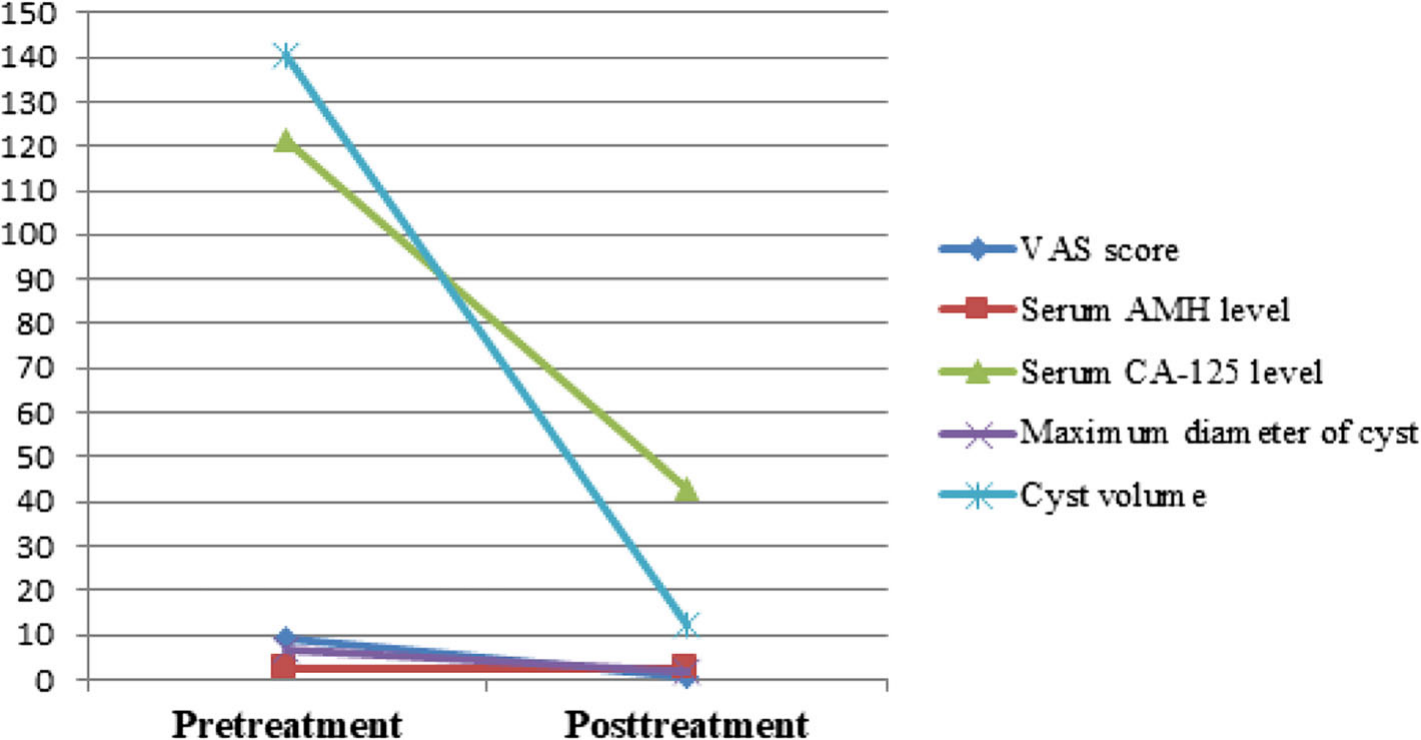
Graph showing changes in all variables after ethanol sclerotherapy. The timepoint of post-treatment indicates the last follow-up, a duration that varied among all study subjects. Note a significant decrease in all variables, excluding serum AMH level, which did not reduce and was associated with preserved ovarian reserve after ethanol sclerotherapy treatment. VAS = Visual Analogue Scale, AMH = anti-Müllerian hormone, CA-125 = cancer antigen 125

**Fig. 5:**
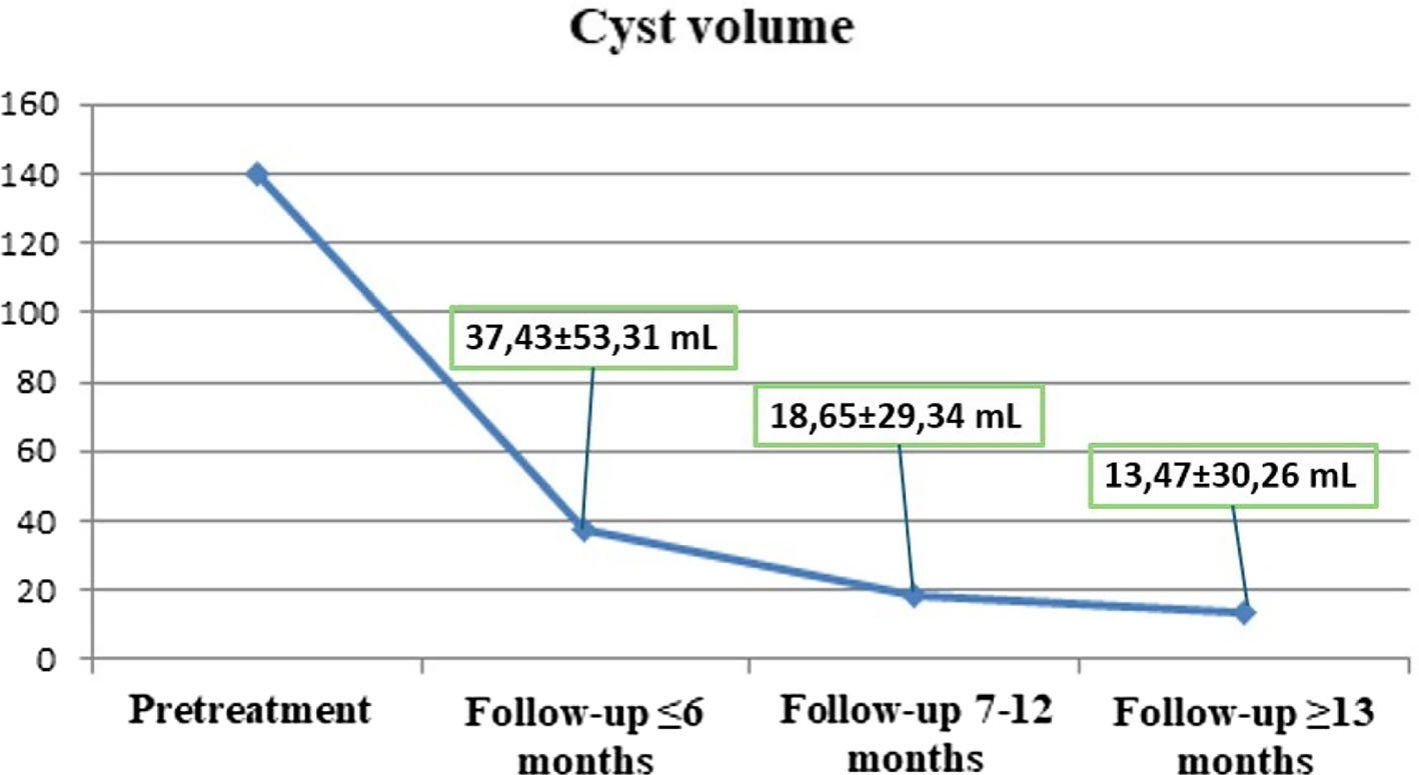
Graph showing changes of cyst volume according to follow-up times. Note the decrease in cyst volumes as the follow-up time increases

There was a significant reduction in cyst size, pain, and serum CA-125 levels (*p* < 0.001). All patients treated for pain experienced pain relief (*n* = 40, *p* < 0.001), with complete pain resolution (VAS = 0) in 75% (*n* = 30) and a significant decrease in pain (VAS = 1–3) in 25% (*n* = 10). Serum AMH levels showed no significant difference between pretreatment and last follow-up values (*p* = 0.822). Overall, pregnancy was observed in eight patients, six of whom were treated for infertility, resulting in a pregnancy rate of 54% (*n* = 6/11; spontaneous *n* = 5, in vitro fertilization *n* = 1). The pretreatment and post-treatment images of two patients are shown in Fig. 3. Table 2, Figs. 4, and 5 summarize the outcomes of ethanol sclerotherapy.

**Table 2:** Outcomes of ethanol sclerotherapy

Variables	Before treatment		After treatment		*p* value
	Mean ± SD	Median (Min–Max.)	Mean ± SD	Median (Min–Max.)	
Maximum diameter of cysts (cm)	6.5 ± 2.4	6.0 (3.2–14.0)	1.8 ± 2.1	0.6 (0.0–7.8)	**< 0.001**^**a**^
Volume of cysts (mL)	140.4 ± 179.8	81.0 (11.0–856.0)	12.0 ± 22.9	0.0 (0.0–119.0)	**< 0.001**^**a**^
VAS score	8.8 ± 2.4	10.0 (0.0–10.0)	0.5 ± 0.9	0.0 (0.0–3.0)	**< 0.001**^**a**^
Serum AMH level (ng/l)	2.3 ± 1.9	1.94 (0.0–7.2)	2.4 ± 2.3	1.7 (0.0–8.0)	0.822^a^
Serum CA-125 level (U/ml)	121.7 ± 217.6	63.30 (4.7–1306.7)	43.1 ± 39.1	30.1 (5.2–182.3)	**< 0.001**^**a**^

Statistically significant values are highlighted in bold within the “*p* value” column

*a* Wilcoxon sign test, *SD* Standard deviation, *Min* Minimum, *Max* Maximum, *VAS* Visual analogue scale, *AMH* Anti-Mullerian hormone, *CA-125* Cancer antigen 125

**Fig. 6:**
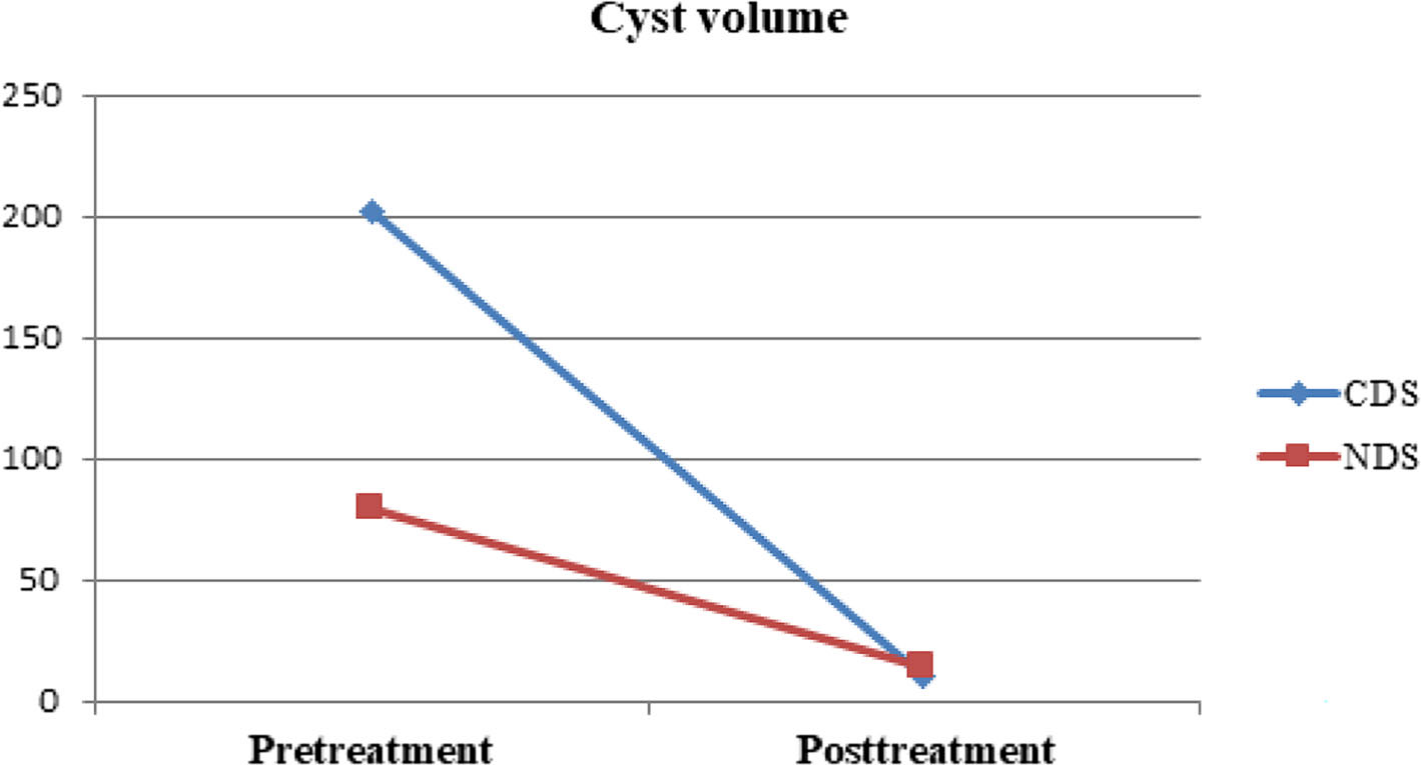
Graph showing changes of cyst volume according to the treatment technique. The decrease in the cyst volume is more significant in CDS group than in the NDS group. The pretreatment cyst sizes are larger in those treated with CDS than NDS, and it was shown that the large pretreatment cyst size is associated with a significant post-treatment size decrease. Furthermore, there is no significant difference between these techniques according to final cyst volume. CDS = catheter-directed sclerotherapy, NDS = needle-directed sclerotherapy

Furthermore, clinical success rates were compared between CDS and NDS, with 50% of cysts treated with each technique (CDS: *n* = 34, NDS: *n* = 34). The decrease in maximum cyst diameter (*p* = 0.021) and cyst volume (*p* = 0.016) was more significant in the CDS group than in the NDS group (Table 3). The pretreatment cyst volumes were larger in those treated with CDS than NDS (*p* = 0.033) (Fig. 6), and the large pretreatment cyst volume was associated with a significant post-treatment size decrease (*p* < 0.001). The cysts disappeared at the last follow-up with a rate of 53% (*n* = 18) in the CDS group and 50% (*n* = 17) in the NDS group, indicating no significant difference between these techniques (*p* = 1.000). There was no difference between these two groups in terms of other variables; see Table 3.

**Table 3:** Comparison of CDS and NDS techniques

Differences in variables before treatment and at last follow-up after treatment	Treatment technique				
	CDS		NDS		*p* value
	Mean ± SD	Median (Min–Max.)	Mean ± SD	Median (Min–Max.)	
Difference in the maximum diameters of cysts (cm)	5.5 ± 3.1	5.0 (0.2–14.0)	4.0 ± 2.1	3.5 (0.9–8.7)	**0.021**^**a**^
Difference in the volumes of cysts (mL)	191.5 ± 227.0	91.6 (10.0–856.0)	65.3 ± 51.4	54.0 (11.0–218.0)	**0.016**^**b**^
Difference in the VAS scores	7.5 ± 3.2	8.0 (0.0–10.0)	9.2 ± 1.0	9.5 (7.0–10.0)	0.163^b^
Difference in the serum AMH levels (ng/l)	0.4 ± 1.5	0.1 (− 2.4–5.3)	-0.1 ± 0.9	− 0.3 (− 2.3–2.6)	0.224^b^
Difference in the serum CA-125 levels (U/ml)	48.7 ± 64.9	32.7 (− 18.0–305.7)	107.2 ± 285.2	15.7 (− 104.8–1294.6)	0.522^b^

Statistically significant values are highlighted in bold within the “*p* value” column

*CDS* Catheter-directed sclerotherapy, *NDS* Needle-directed sclerotherapy, *a* Student-t test, *b* Mann–Whitney U test, *SD* Standard deviation, *Min* Minimum, *Max* Maximum, *VAS* Visual analogue scale, *AMH* Anti-Mullerian hormone, *CA-125* Cancer antigen 125

**Fig. 7:**
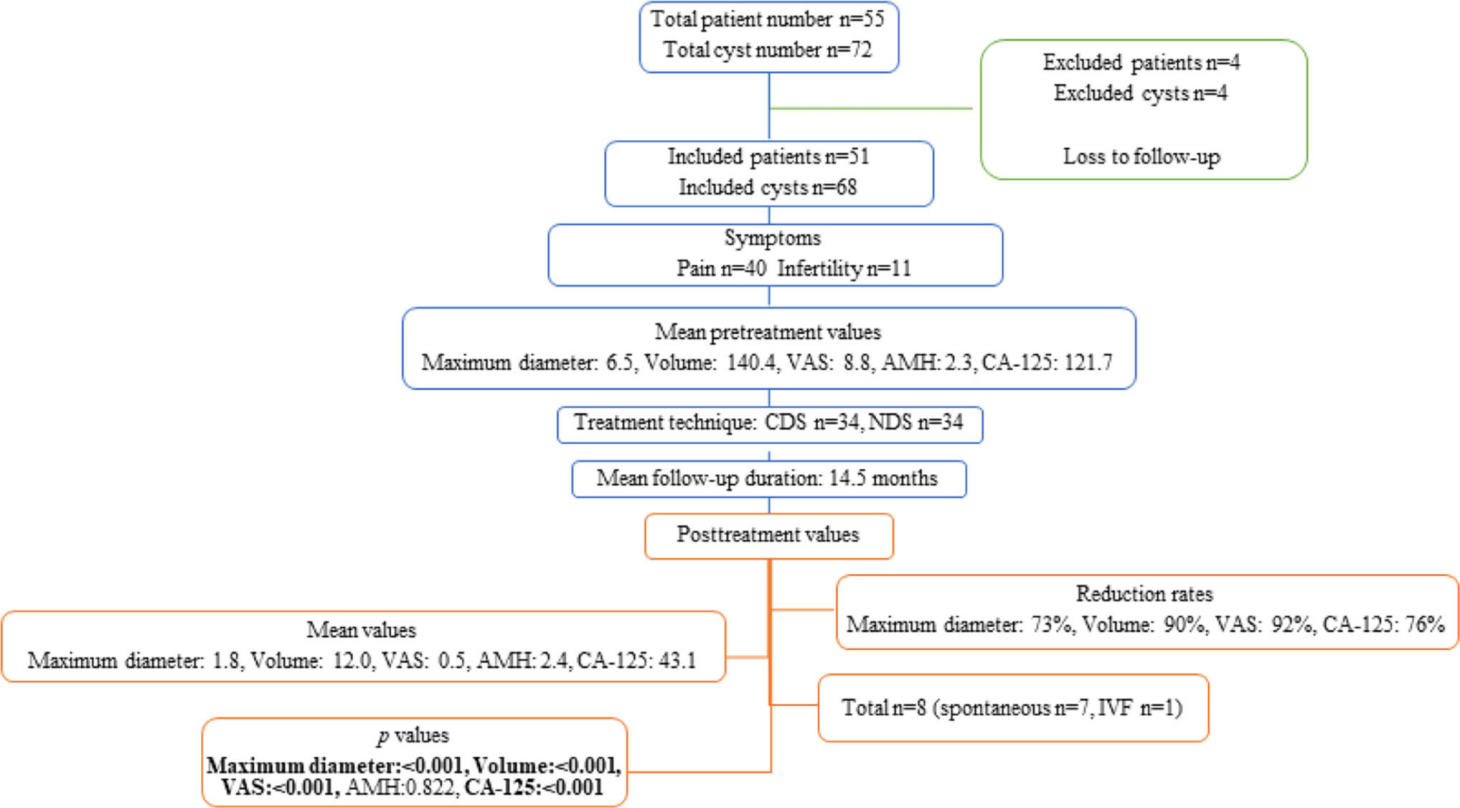
Flowchart summarizing the selection of participants for the study, and results of the study. VAS = Visual Analogue Scale, AMH = anti-Müllerian hormone, CA-125 = cancer antigen 125, CDS = catheter-directed sclerotherapy, NDS = needle-directed sclerotherapy, IVF = in vitro fertilization

The selection of participants for the study and results of the study are summarized as flowchart in Fig. 7.

## Discussion

This retrospective study provides key factors that should be considered in the decision-making process of choosing CDS or NDS, including cyst size, cyst location, cyst viscosity, and tissue rigidity, based on our single-center experience. Since cyst viscosity and tissue rigidity are unpredictable before the procedure, the decision on CDS or NDS was made during the procedure. Half of the cysts were treated with CDS and another half with NDS. There were significant reductions in maximum cyst size, pain, and serum CA-125 levels in all patients (*p* < 0.001), with no decrease in serum AMH levels (*p* = 0.822). CDS showed a greater reduction in cyst sizes compared to NDS (*p* < 0.05). Pretreatment cyst sizes were larger in the CDS group (*p* = 0.033), correlating with a more pronounced post-treatment size decrease (*p* < 0.001), suggesting that the larger pretreatment cyst size in the CDS group may contribute to the greater reduction in cyst sizes.

In this study, both treatment techniques were associated with high clinical success. One patient with two cysts initially treated with NDS experienced recurrence, which was successfully treated with CDS in the second session. In the study of Noma and Yoshida [13], recurrence rates were 62.5%, 9.1%, and 3.8% in groups treated with ethanol instilled for < 10 min, > 10 min, and laparoscopic cystectomy, respectively, indicating significant differences in recurrence rates based on the timing of ethanol sclerotherapy. In our study, the timing for sclerotherapy was consistently set at 15 min. Additionally, in contrast to our approach, they did not apply irrigation during NDS, possibly leading to higher recurrence rates compared to surgical intervention.

Ethanol sclerotherapy offers a critical advantage in preserving ovarian reserve compared to surgery, which inevitably leads to a decrease in ovarian tissue due to adjacent tissue removal or electrocoagulation [5, 14–23]. Roman et al. [22] demonstrated significant ovarian tissue removal during surgery, proportionate to cyst size. However, in sclerotherapy, solely endometrioma is targeted; that is why normal ovarian tissue is not damaged, and serum AMH levels do not decrease [4, 24–27]. Vaduva et al. [28] compared NDS with laparoscopic cystectomy, showing a significant decrease in AMH levels in the latter group. Nevertheless, a recent meta-analysis [29] found no statistically significant differences in recurrence and pregnancy rates between surgery and sclerotherapy groups, although this review included various sclerosing agents such as tetracycline besides ethanol. In our study, all sessions utilized 95% sterile ethanol sclerotherapy, chosen over surgery unless malignancy was suspected, with the final decision made during a multidisciplinary forum involving gynecologists and interventional radiologists.

The main limitation of this study was its retrospective design. Furthermore, we did not compare ethanol sclerotherapy with other treatment techniques, including surgery with or without hormonal therapy. Therefore, further studies are needed to compare both CDS and NDS with surgical techniques. Other significant limitations were a relatively short follow-up period (14.5 months) and a small sample size (51 patients, 68 cysts).

In conclusion, both catheter- and needle-directed ethanol sclerotherapy are effective methods preserving ovarian reserve in endometrioma treatment if the proper technique is applied. The choice between CDS and NDS should be determined during the procedure, with a preference for CDS when feasible. Crucial factors in making this decision include cyst size, cyst location, viscosity of cyst content, and tissue rigidity. It is essential to rule out malignancy using subtracted contrast-enhanced T1-weighted images before proceeding with ethanol sclerotherapy.

### Supplementary Information

Below is the link to the electronic supplementary material.

Supplementary Video 1 CDS procedure. A 24-year-old patient with left ovarian endometrioma complaining of infertility was treated with CDS. In the 6-month follow-up, the cyst regressed significantly, and the patient got pregnant spontaneously
